# Neonatal Resuscitation in Children 2021: Focus on Training, Technology, and New Clinical Approaches

**DOI:** 10.3390/children9020175

**Published:** 2022-02-01

**Authors:** Daniele Trevisanuto, Viraraghavan Vadakkencherry Ramaswamy, Paolo Ernesto Villani

**Affiliations:** 1 Department of Woman’s and Child’s Health, University Hospital of Padua, 35128 Padua, Italy; 2 Department of Neonatology, Ankura Hospital for Women and Children, Hyderabad 500072, India; 19.vira@gmail.com; 3 Department of Pediatrics, Fondazione Poliambulanza Istituto Ospedaliero, 25124 Brescia, Italy; paolo.villani@poliambulanza.it

Neonatal resuscitation remains a hot topic for pediatricians and neonatologists worldwide. Last year, several articles on different aspects related to neonatal resuscitation were published in the journal Children. The topics covered included staff training [1,2], technological aspects [3,4,5,6], and the investigation of “new” clinical interventions [7,8,9,10,11,12].

Amongst the various articles published last year in Children, that of Bettinger et al. reviewed the available literature on the topic of training opportunities during neonatal resuscitation [1]. The article’s title, “Making every birth a learning event”, mirrors the aim of the review, which was to explore the teaching opportunities that arise whenever a newborn is delivered. Bettinger et al. rightly point out that there are two crucial periods during which health personnel may be trained on neonatal resuscitation while attending a delivery. The first is during the delivery, at which time a trainee may gain expertise through various methods, including the traditional in-person teaching, remote coaching, or the use of automated guidance based on available software dedicated to neonatal resuscitation. The second training opportunity occurs after the delivery of the neonate, when the team has time to engage in a well-structured debriefing session conducted by an expert in the field. The review underlines the strengths, the limitations, and the gaps in knowledge of such educational approaches in relation to the setting and the available resources (high- vs. low-resource settings). Another interesting article by Haynes et al. from Norway suggests that low-dose, high-frequency in situ simulation improves staff performance [2]. These claims are based on previous reports that showed that low-dose, high-frequency training after participation in an initial course on neonatal resuscitation contributed to improving the initiation as well as timeliness of resuscitation procedures, which also translated into reduced 24-h neonatal mortality in low-resource settings [13,14]. Although these findings seem promising, the content to be repeated across recurrent training sessions as well as the ideal frequency of these sessions is yet to be established with good certainty.

Amongst the many articles published in Children, four of them describe emerging technologies for improving neonatal care at birth [3,4,5,6]. Two of them reported on new models of trolleys that could aid in the resuscitation of neonates with intact placental circulation at the bed side of the mother [3,4]. While one study was conducted in a high-resource setting, the other one described a low-cost trolley which could be used in low-resource settings. Though these new pieces of equipment offer all the required technical support to appropriately manage a neonate in need of resuscitation/stabilization at birth, the clinical impact of this approach needs to be established. Heart rate is the most important clinical indicator monitored during neonatal resuscitation [15]. Several modalities to monitor heart rate have been reported over the past few years [16]. In their clinical study, Rettedal et al. compared heart response from a dry-electrode ECG, the 3-lead ECG, and the pulse oximetry during newborn resuscitation [5]. The dry-electrode ECG was faster and more effective in providing a valuable HR than the 3-lead ECG and the pulse oximeter during the resuscitation of 48 neonates. Mannequin studies conducted in high- and low-resource settings have shown that tools capable of providing ventilator feedback (i.e., mask leak, peak inspiratory pressure, and tidal volumes) improve the quality of positive pressure ventilation deployed by healthcare givers [17,18]. A study by Haynes et al. compared ventilation parameters derived from a new, low-cost, high-fidelity simulator with those from real neonates in a group of experienced pediatricians [6]. Although the authors reported some limitations, the mannequin was able to simulate real life clinical situations. They concluded that this simulator could be an effective instrument for teaching positive pressure ventilation in neonates with mild as well as severe lung disease at birth. New technology that could aid in optimizing the healthcare providers’ training and enhance neonatal monitoring immediately after delivery have increased exponentially over the last few years. Further research is needed to understand if these promising tools will impact the short and long-term clinical outcomes of neonates.

The ideal initial fraction of inspired oxygen (FiO_2_) in preterm infants needing resuscitation at birth remains a matter of debate [15,19]. Amongst the published studies in Children in 2021, one human and one animal study have each assessed the impact of initial FiO_2_ or targeting different SpO_2_ ranges in two different clinical contexts [7,8]. The pilot study by Law et al. published in the journal compared two initial FiO_2_ values (30% vs. 60%) in 30 infants born at 23–28 weeks’ gestation requiring delivery room resuscitation, with the aim of evaluating the feasibility of conducting such a trial [7]. Feasibility was defined by the authors in terms of achieving an adequate post-intervention consent rate and achieving a meaningful difference in the cumulative supplied oxygen in the first 5 min. The results of this feasibility study are the basis for a larger randomized controlled trial (https://clinicaltrials.gov/ct2/show/NCT02858583, accessed on 2 January 2022). A pre-clinical study on a similar subject, but in a different context, assessed the impact of low (85–94%) vs. high (95–99%) SpO_2_ ranges on the ductal flow during and after resuscitation in lambs with meconium aspiration syndrome and persistent pulmonary hypertension [8]. In the immediate post-resuscitation phase, higher SpO_2_ targeting resulted in a faster reversal of the shunt to left-to-right direction across the patent ductus arteriosus, leading to improved pulmonary flow; however, this advantage was not maintained at 60 min.

The aspects related to the outcome of peri-viable infants born at 22–25 weeks’ gestational age was reported in a well-written review [9] and an observational single center cohort study from Taiwan [10]. The authors of the latter study reported a significant increase in the survival of peri-viable extremely low-gestational age infants after the implementation of the 2015 American Academy of Pediatrics Neonatal Resuscitation Program, which advocated a change in the threshold of viability from 23 weeks’ to 22 weeks’ gestation. However, data on long-term follow up of these high-risk infants are needed before drawing conclusive prognostic information.

Finally, two interesting articles have added information and offered hypotheses on patient management during the advanced phase of neonatal resuscitation. Kim et al. extensively reviewed the existing experimental and clinical evidence on chest compressions along with sustained inflation during cardiopulmonary resuscitation in neonates [11]. The findings suggest that this new approach is likely to be more effective than the currently used 3:1 compression to ventilation ratio in terms of a shorter time to the return of spontaneous circulation (ROSC), decreased mortality, and improvement in other respiratory and hemodynamic parameters. Results from a large multicenter randomized controlled trial (SURV1VE-trial-https://clinicaltrials.gov/ct2/show/NCT02858583, accessed on 2 January 2022) are expected in 2024. Sankaran et al. compared the effect of two flush volumes (1 mL vs. 2.5 mL) of normal saline in severely asphyxiated lambs immediately after epinephrine administration [12]. After the first dose of epinephrine, the ROSC was more frequently achieved in the group that received 2.5 mL compared to the group treated with 1.0 mL normal saline flush (80% vs. 43%; *p* = 0.08). The median time to ROSC and cumulative epinephrine dose were similar between the two groups. Although interesting, these findings need to be confirmed in a well-designed randomized, controlled trial.

In conclusion, although there has been great progress over the last decade in our understanding of the pathophysiology of infants in need of resuscitation at birth, gaps in knowledge remain for a range of aspects related to neonatal resuscitation. The articles published in Children in 2021 have contributed immensely in furthering our knowledge in relation to neonatal resuscitation, and many of them could be the bases for future clinical trials.

## Abbreviations

ECG	Electrocardiogram
FiO_2_	Fraction of inspired oxygen
ROSC	Return of spontaneous circulation
